# Biogeography and Character Evolution of the Ciliate Genus *Euplotes* (Spirotrichea, Euplotia), with Description of *Euplotes curdsi* sp. nov.

**DOI:** 10.1371/journal.pone.0165442

**Published:** 2016-11-09

**Authors:** Mitchell J. Syberg-Olsen, Nicholas A. T. Irwin, Claudia Vannini, Fabrizio Erra, Graziano Di Giuseppe, Vittorio Boscaro, Patrick J. Keeling

**Affiliations:** 1 Canadian Institute for Advanced Research, Department of Botany, University of British Columbia, Vancouver, British Columbia, Canada; 2 Dipartimento di Biologia, Università di Pisa, Pisa, Italy; Laboratoire de Biologie du Développement de Villefranche-sur-Mer, FRANCE

## Abstract

Ciliates comprise a diverse and ecologically important phylum of unicellular protists. One of the most specious and best-defined genera is *Euplotes*, which constitutes more than 70 morphospecies, many of which have never been molecularly tested. The increasing number of described *Euplotes* taxa emphasizes the importance for detailed characterizations of new ones, requiring standardized morphological observations, sequencing of molecular markers and careful comparison with previous literature. Here we describe *Euplotes curdsi* sp. nov., distinguishable by the combination of the following features: 45–65 μm length, oval or elongated shape with both ends rounded, narrow peristome with 25–34 adoral membranelles, conspicuous paroral membrane, double-*eurystomus* dorsal argyrome type, 6–7 dorsolateral kineties and 10 frontoventral cirri. Three populations of the novel species have been found in brackish and marine samples in the Mediterranean and the White Sea. We provide the SSU rRNA gene sequences of these populations, and an updated phylogeny of the genus *Euplotes*. Using the molecular phylogenetic tree, we inferred aspects of the biogeographical history of the genus and the evolution of its most important taxonomic characters in order to provide a frame for future descriptions. Ultimately, these data reveal recurrent trends of freshwater invasion and highlight the dynamic, yet convergent, morphological evolution of *Euplotes*.

## Introduction

Ciliates (unicellular protists of the phylum Ciliophora) are ubiquitous, abundant and eye-catching components of aquatic environments [1–4]. Yet despite their large diversity and ecological importance, only a handful of ciliates have been intensively studied after their original description. These model species, including those belonging to the genera *Paramecium* [5–7] and *Tetrahymena* [8–10], are easy to grow under laboratory conditions and relatively common in the environment. Another model ciliate is the hypotrichous *Euplotes* (Spirotrichea, Euplotia), which has been used to study mating types and sexual pheromones [11–13], adaptations to cold temperatures [14, 15], the geographical distribution of unicellular organisms [16], and intracellular bacterial symbioses [17–20].

Even within the model genera, some species or even strains are much more intensely studied than others making species identification vital for both experimental reproducibility and interpretation. Indeed, the topic of species recognition itself has underlined much of the literature on ciliate diversity [21–23], and there are many reports of discrepancies between morphological and molecular data [24, 25]. *Euplotes* is a comparatively straightforward case: despite the number of recognized species (over 70), a good set of quantifiable morphological characters allows taxonomists to define appropriate morphospecies boundaries. Although some ambiguous situations exist (e.g. [26–28]), it is generally easy to perform identifications using standardized techniques, and molecular data tend to corroborate the validity of morphologically-defined species [26, 29]. However, with the growing number of novel taxa being reported [30–36], it is increasingly important for descriptions to be accurate and include multiple types of data that are thoroughly compared to all relevant literature. In *Euplotes*, many molecular marker sequences have been ascribed to morphospecies without providing morphological results supporting the identification, and those sequences are routinely used as barcodes for identification. Even more commonly, new species characterizations are based on a single population, which does not provide information on the intraspecific variability.

In this paper we describe a new species, *Euplotes curdsi* sp. nov., represented by three geographically separated populations and characterized both morphologically and molecularly, by sequencing the SSU rRNA gene. Because the last extensive monography on the genus *Euplotes* [37] is missing many of the recently reported species and predates the use of molecular systematics, we also provide a phylogeny of the genus based on current data. This summary of biogeography and character evolution should help future studies identifying new species (or redescribing old ones) put them in the correct evolutionary and ecological frame.

## Materials and Methods

### Sampling and culturing

**Fig 1:**
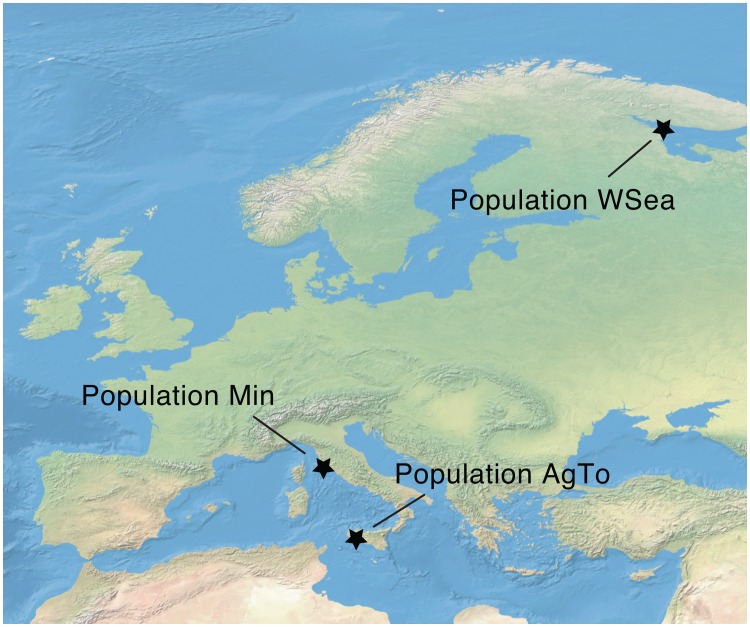
Sampling locations of the three described populations of *Euplotes curdsi* sp. nov. The species has been observed in a brackish lagoon in Italy as well as in marine sediments from the Mediterranean and White Sea.

The three investigated populations of *Euplotes curdsi* sp. nov. were collected in shallow sediments from the brackish (12‰ salinity) Orbetello Lagoon in Italy (population Min) and marine sites in Agrigento, Italy (32‰ salinity) and Sredny Island, Russia (25‰ salinity) (populations AgTo and WSea respectively [38]) (Fig 1). No specific permissions were required for sampling in these locations, and the field studies did not impact or involve any endangered or protected species. Monoclonal strains (Min1, AgTo2, AgTo3, Wsea4, WSea5 and WSea6) were then established and cultured in artificial 5‰ or 32‰ salinity water, maintained in a 19–20°C incubator with a 12:12 light/dark cycle and fed with the green alga *Dunaliella tertiolecta*.

### Morphological characterization

Specimens were characterized using a combination of differential interference contrast (DIC) microscopy, silver nitrate staining (Chatton-Lwoff method [39]), Feulgen staining, fluorescent microscopy of DAPI stained living and fixed cells and scanning electron microscopy (SEM). A minimum of 10 measurements per strain, and 15 per population, were collected for each character. DIC and fluorescent microscopy was performed using a Zeiss Axioplan 2 upright microscope mounted with a Zeiss Axiocam 503 color digital camera. Cellular features were measured using the computer program Macnification v2.0.5 (Orbicule bvba). The wet silver stain method was employed to visualize the argentophilic cortical structures (“silverline” or “argyrome”). Nuclear features were observed through the Feulgen staining procedure, and *in vivo* by keeping cells in an 8 μg/mL DAPI infused culture solution in the dark for 30 minutes prior to wet mounting on slides [40]. Formaldehyde fixed Min1 cells were also treated with SlowFade^®^ Gold antifade reagent with DAPI (Life Technologies) as a part of the FISH hybridization protocol [41]. For SEM, cells were fixed with 4% OsO_4_, attached to slides with 0.01% poly-L-lysine solution (Electron Microscopy Sciences) and gradually dehydrated with ethanol before being subject to critical point drying and subsequent 4 nm coating with an 80/20 mixture of platinum and palladium. Observations were performed on a Hitachi S-4700 microscope.

### SSU rRNA gene sequencing

AgTo and WSea strains were sequenced as detailed elsewhere [38]. Prior to DNA extraction, a Min1 subculture was briefly starved. Cells were then hand-picked using a glass micropipette, washed multiple times in sterile water, and fixed in 70% ethanol. DNA extraction was performed using the NucleoSpin^™^ Plant II DNA extraction kit (Macherey-Nagel, Düren, Germany). The primers used for PCR amplification were 18S F9 Euk (5’-CTGGTTGATCCTGCCAG-3’) and 18S R1513 Hypo (5’-TGATCCTTCYGCAGGTTC-3’) [42]. DNA amplification was done in a C1000^™^ Thermal Cycler (BioRad) according to the following thermal profile: 3 minutes at 94°C, followed by 35 cycles each consisting of 30 s at 94°C, 30 s at 55°C and 2 minutes at 72°C, ending with 5 minutes at 72°C. PCR products were sequenced using Sanger sequencing by AGOWA GmbH (Berlin, Germany) with internal primers 18S F783 (5’-GACGATCAGATACCGTC-3’), 18S R536 (5’-CTGGAATTACCGCGGCTG-3’) and 18S R1052 (5’-AACTAAGAACGGCCATGCA-3’) [42]. Electropherograms were visualized using 4Peaks v1.8 (nucleobytes.com) and assembled to obtain the complete gene sequence.

### Phylogenetic analysis

*Euplotes* sequences were collected from the GenBank/EMBL databases using the advanced search function to return all *Euplotes* SSU rRNA gene sequences. Results were then narrowed to only include sequences longer than 1500 nucleotides, and 11 additional sequences were removed due to poor quality or potential misidentification. Two data sets were derived from the remaining 164 *Euplotes* sequences, one including all sequences and a second consisting of 46 sequences, individually selected as a representative of each species. We made exceptions in the case of *Euplotes encysticus* where a large intraspecific variability was observed, and *Euplotes charon* where the two remaining sequences did not cluster with each other. Each data set also includes 14 outgroup sequences belonging to the related genera *Aspidisca*, *Certesia*, *Euplotidium* and *Gastrocirrhus*. Mafft v7.294b [43] was used to align the sequences. The character matrices were inspected using ClustalX v2.1 [44], and columns at both ends were removed if containing more than 60% missing data. Maximum Likelihood trees were inferred for both data sets with IQ-TREE v1.4.2 [45] using the GTR+I+G4 model chosen by AIC and BIC criteria. 100 and 1000 bootstrap pseudoreplicates were calculated for the large and small data sets respectively. Bayesian Inference analyses were performed with MrBayes v3.2.5 [46] using the same nucleotide substitution model, iterating for 1,000,000 generations on 3 independent runs, each consisting of 1 cold and 3 heated chains.

### Data availability

The newly obtained SSU rRNA gene sequences of *E*. *curdsi* are available from the GenBank/EMBL databases (accession numbers: Min1, LT615048; AgTo3, KX819312; WSea4, KX819313; WSea6, KX819314). Strains AgTo2 and WSea3 were previously deposited with the provisional name of “*E*. *vannuccii*” (accession numbers: EF094971-2). A living culture of type strain Min1 has been deposited at the Culture Collection of Algae and Protozoa (CCAP 1624/28). Slides with fixed specimens are available from the Beaty Biodiversity Museum at the University of British Columbia (Vancouver, Canada; accession numbers: A92521-2). Holotype and paratypes were marked as in Foissner et al [47].

### Nomenclatural Acts

The electronic edition of this article conforms to the requirements of the amended International Code of Zoological Nomenclature, and hence the new names contained herein are available under that Code from the electronic edition of this article. This published work and the nomenclatural acts it contains have been registered in ZooBank, the online registration system for the ICZN. The ZooBank LSIDs (Life Science Identifiers) can be resolved and the associated information viewed through any standard web browser by appending the LSID to the prefix “http://zoobank.org/”. The LSID for this publication is: urn:lsid:zoobank.org:pub:F8968526-1105-46D9-A014-EF832107108E. The electronic edition of this work was published in a journal with an ISSN, and has been archived and is available from the following digital repositories: PubMed Central, LOCKSS. The LSID for *Euplotes curdsi* sp. nov. is urn:lsid:zoobank.org:act:05E0ECEC-07CF-4D9B-951C-C157BCE349B7.

## Results

### Morphological description of *Euplotes curdsi* sp. nov. (Fig 2)

**Fig 2:**
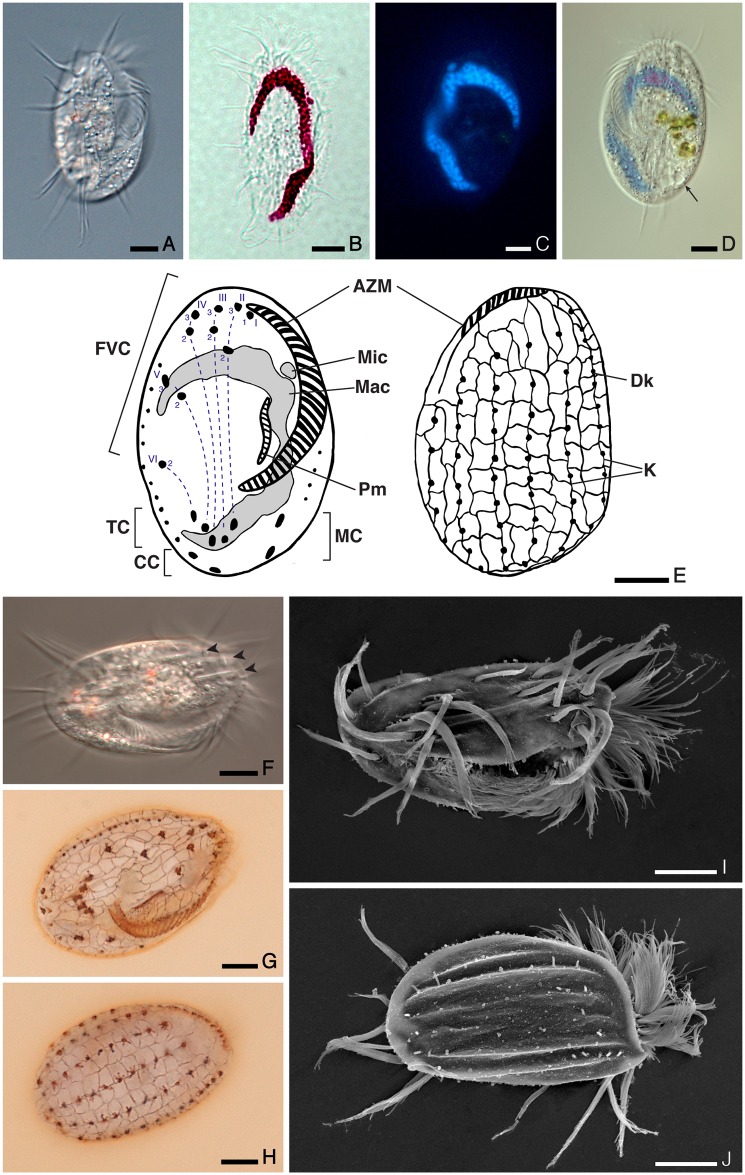
Morphology of *Euplotes curdsi* sp. nov., as represented in specimens of the type strain Min1. **(A)** Ventral view of a living organism. **(B)** Nuclear apparatus stained with the Feulgen method. **(C)** Nuclear apparatus stained by DAPI in a living organism. **(D)** Superimposed differential interference contrast and fluorescence micrographs of the same specimen. **(E)** Schematics of argyrome and nuclear features observable on the ventral (left) and dorsal (right) side, drawn from fixed cells (paratypes P_1_ and P_2_) with a tracing device. The Wallengreen [48] numerical system for frontoventral cirri numeration is also shown. **(F)** Ventral view of a living organism, showing the prominent ventral ridges. **(G)** Ventral argyrome features after silver staining (holotype). **(H)** Dorsal argyrome features after silver staining (paratype P_3_). **(I)** Ventral view of a cell using scanning electron microscopy (SEM). **(J)** Dorsal view of a cell using SEM. Scale bars represent 10 μm. A small arrow points at the contractile vacuole. Black arrowheads indicate the position of ventral ridges. AZM, adoral zone of membranelles; CC, caudal cirri; Dk, dikinetid; FVC, frontoventral cirri; K, dorsolateral kineties; Mac, macronucleus; MC, marginal cirri; Mic, micronucleus; Pm, paroral membrane; TC, transverse cirri.

The average cell size is 53.9 ± 5.0 x 31.2 ± 3.9 μm in silver stained specimens of the three populations (Fig 2G and 2H). The strain Min1 was also measured *in vivo* (57.1 ± 5.0 x 30.4 ± 3.0 μm) (Fig 2A), allowing an estimate of size reduction due to fixation of about 6–9%. Size did not significantly differ between well-fed and starved Min1 cells. Cell shape is oval to ellipsoid (Fig 2A), with a length:width ratio of about 1.6–1.9, lower in the marine populations and higher in the brackish Min1 strain. The left side is slightly more curved than the right side, and both ends are rounded. No notch or protrusion at the anterior end was observed. The peristome is narrow but long and deep, extending for about 65–75% of body length (Fig 2A, 2F and 2I). There are 25–34 membranelles in the adoral zone (AZM), starting at the top of the cell (where no peristomial collar is visible) and continuing down the left side in a regular curve that occasionally becomes more angular in proximity to the cytostome (Fig 2G). The contractile vacuole is often not visible, but appears circular and prominent in the bottom right area of stressed cells (Fig 2D). The dorsal argyrome (dargyrome) is of the double-*eurystomus* type, with two equal rows of roughly rectangular alveoli between each pair of dorsolateral kineties (Fig 2H). Kineties are six or (more commonly) seven, and the mid-dorsal row contains up to 10–12 dikinetids (Fig 2H). There are always 10 frontoventral and five transverse cirri, plus four (rarely, three) well developed caudal cirri, 2 of which are usually positioned to the left of the peristome (marginal cirri) (Fig 2G and 2I). The paroral membrane is conspicuous in silver stained specimens (Fig 2G), but the corresponding cilia rarely protrude outside the buccal cavity (Fig 2I). The dorsal side of the cell is poorly decorated, with inconspicuous ridges slightly more pronounced near the margins (Fig 2J). The ventral side is instead heavily sculptured, with three thin but very prominent ridges clearly visible in living organisms, and shorter furrows in correspondence to the transverse cirri (Fig 2F and 2I). The most conspicuous ridge starts at the very anterior end, passes between frontoventral cirri III/3 and IV/3 and extends just beyond the transverse cirri. The macronuclear shape is somewhat variable but in most cases it is “C” shaped or slightly “3” shaped, with irregularly dense chromatin (Fig 2B and 2C). Often one or both ends of the C appear wider, while the central part of the macronucleus is thinner and faintly stained by DAPI. The single micronucleus is large but sometimes not visible, closely associated to or lodged in an anterior depression of the macronucleus (Fig 2B and 2C).

### Other descriptive features

The brackish Min1 strain, sampled in lagoonal sediments at 12‰ salinity, was able to grow in a salinity range of 5–20‰, but died after less than a week when moved to media with higher or lower salinities. This strain was also resistant to long starvation periods (no new algal inoculations for up to two months) and generally required less food than most other *Euplotes* cultures in our collection. No cyst was ever observed.

No endosymbiont could be detected in living or FISH observations of the Min1 strain (data not shown).

### Molecular data and phylogeny

The SSU rRNA gene sequences of all monoclonal strains in the three collected populations were identical. The best 100 hits in BLASTn analysis (ignoring sequences from uncultured organisms) belong to the *Euplotes* genus, with *E*. *nobilii* strain AC4 being the best hit (accession number: EF094969; 97.4% similarity).

**Fig 3:**
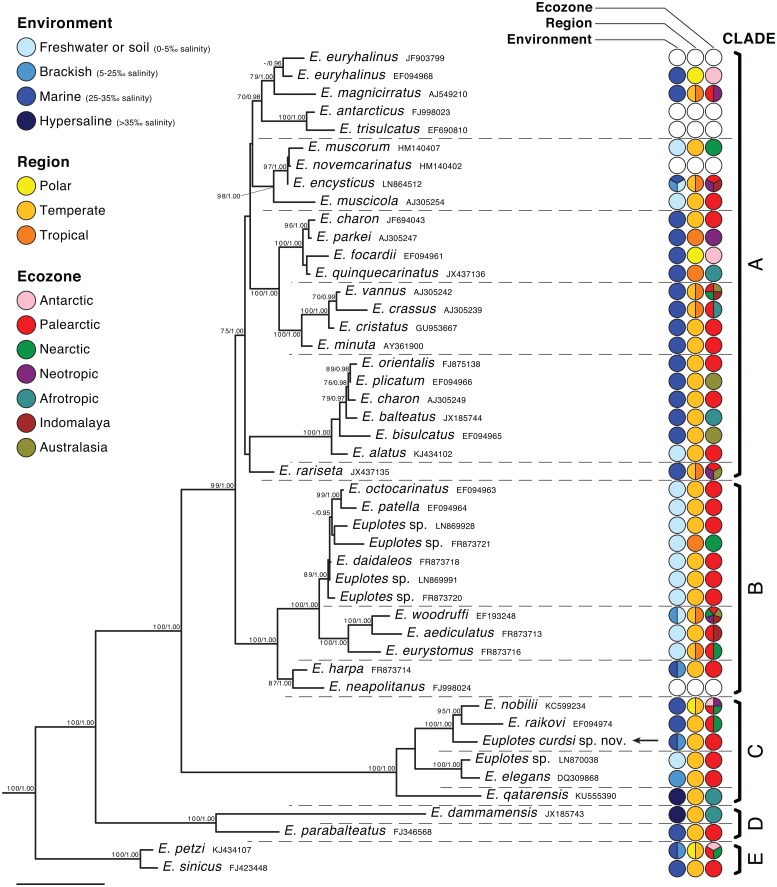
Maximum Likelihood phylogenetic tree of the genus *Euplotes* based on SSU rRNA gene sequences (46-sequence dataset, 2140 characters). The outgroup used to root the tree has been omitted. Numbers associated with nodes represent bootstrap/posterior probability; values below 70/0.95 are not shown. Labeled nodes were recovered by all methods and datasets tested (see Materials and Methods). Five major clades are identified by capital letters (A-E). Dashed lines separate 15 smaller subclades. Data on environment, region and ecozone were obtained from published papers or GenBank metadata associated with all available sequences for each species (see also S1 Fig). Unknown information is displayed as empty circles. The arrow points at the representative sequence of *Euplotes curdsi* sp. nov. The bar represents an inferred evolutionary distance of 5%.

Maximum Likelihood and Bayesian phylogenetic inferences performed on the 46-sequence dataset differed at only two nodes that were weakly supported by both. Similarly, the inter-species topologies obtained by the two methods applied to the 164-sequence dataset did not significantly differ. More discrepancies (4–7 nodes) could be found comparing trees calculated on the two different datasets. However, none of the differences were strongly supported by bootstrap or posterior probability values. Most nodes of the topology were thus recovered by all trees with high support, as shown in Fig 3.

**Fig 4:**
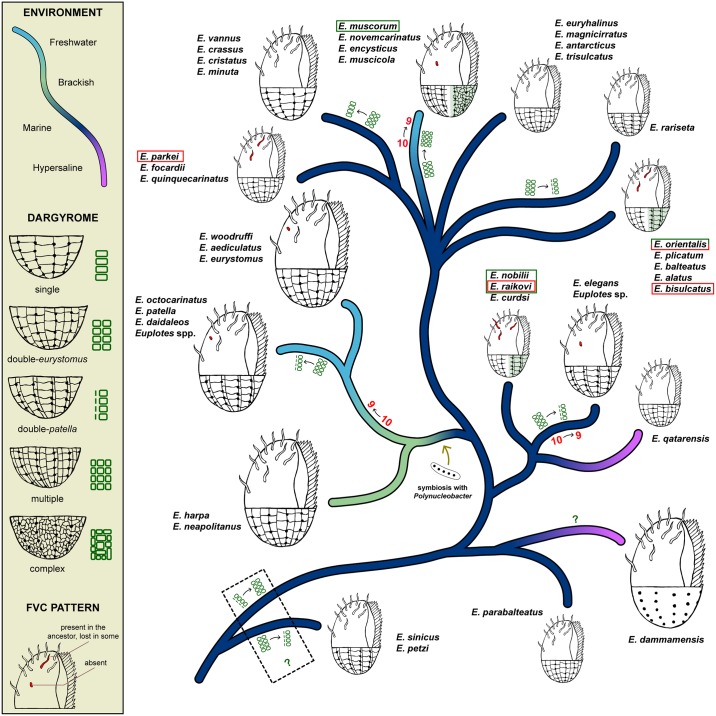
Evolutionary history of the genus *Euplotes*. Phylogenetic relationships are based on recent SSU rRNA gene analyses (see also Fig 3). Colors on branches represent habitats, according to sequence metadata and literature. The 38 morphospecies included were grouped in 15 monophyletic subclades. When species within a subclade did not all share the same environment (see text), majority-rule consensus was applied. Schematic drawings represent the dorsal argyrome type(s) and frontoventral cirral pattern(s) of the species in each subclade (the argyrome type of *E*. *dammamensis* is unknown). Dimensions of the drawings correlate with the average size of the species. The character status of ancestors was inferred using a parsimony criterion. Changes in the argyrome type (green) or the number of frontoventral cirri (red) are shown on branches. Intra-subclade variability is highlighted with colored boxes: red-shaded cirri were lost and green-shaded dargyrome types were acquired by the marked species. The establishment of the obligate symbiosis between *Euplotes* species and the betaproteobacterium *Polynucleobacter* is also shown.

Five major, strongly supported clades can be identified within the genus *Euplotes*, and their phylogenetic relationships reliably inferred. The topology of species within each clade is reasonably supported only in four of them (clades B, C, D and E). Clade A, the most species-rich, includes several non-supported nodes, resulting in not fully resolved relationships among its subclades. A synopsis tree based on supported subclades, and illustrating a consensus of the evolutionary history of the genus *Euplotes* (see also [26, 36, 49]), is shown in Fig 4. Here, *E*. *charon* was removed because it is currently represented by three unrelated SSU rRNA sequences (accession numbers: AF492705, AJ305249, JF694043), none of which are accompanied by morphological descriptions (we removed sequence AF492705 from the phylogenetic analysis, as it most likely belongs to *E*. *magnicirratus*). Until the sequence of a well-characterized *E*. *charon* population is available, the phylogenetic position of this species will remain ambiguous.

*Euplotes curdsi* sp. nov. clusters within clade C. It is the sister group of the marine species *E*. *nobilii* and *E*. *raikovi*. Other close relatives are *E*. *elegans*, the recently characterized *E*. *qatarensis* [36] and an undescribed freshwater population [50].

### Biogeography and character evolution of the genus *Euplotes*

Fig 3 maps environments, latitudinal regions and ecozones for each species. Metadata were obtained only from GenBank information linked with deposited sequences and the associated articles. In this way, the relationship between phylogeny and biogeography is independent from potential identification errors. Several taxa are represented by only a single sequence (see also S1 Fig), but most species sequenced by multiple studies were reported from different ecozones and often latitudes. The variability within subclades is even higher. In contrast, species and subclades tend to be more uniform in terms of habitat (water salinity). Many recent descriptions including molecular sequences state the actual salinity of samples, so we defined environments accordingly (freshwater: 0–5‰; brackish: 5–25‰; marine: 25–35‰; hypersaline: >35‰). The pattern is also summarized in Fig 4, which integrates sequence-associated data with information from all literature. Since salinity was not commonly reported in older papers, habitats are here less strictly defined.

Species in each monophyletic subclade identified in Figs 3 and 4 are largely uniform in terms of frontoventral cirral pattern and dargyrome type. Exceptions are marked in Fig 4. The correlation between these characters and phylogeny is lost when more inclusive assemblages are considered, as subclades with similar features do not necessarily cluster together. Using parsimony, it can be inferred that the ancestor of all known *Euplotes* species had 10 frontoventral cirri and a “double” dargyrome. The prevalence of the double-*eurystomus* pattern (with approximately equal rows of polygons between dorsolateral kineties) suggests that this was the ancestral condition, but the early branching position of *E*. *petzi* and *E*. *sinicus*, species with a double-*patella* pattern [33, 49], questions this assumption. The ancestor of all remaining species most likely had a double-*eurystomus* pattern, however. Including intra-subclade variability, a minimum of seven events of cirri loss (single or multiple) and nine events of dargyrome type change occurred in the evolutionary history of the genus.

## Discussion

### Comparison with similar species

The combination of size (45–65 μm), dargyrome type (double-*eurystomus*), cirral pattern (10 frontoventral cirri) and dorsolateral kinety number (6–7) is unique to *Euplotes curdsi* sp. nov. and sufficient to classify it as a novel taxon. There are, however, five other morphospecies in the same dargyrome and frontoventral cirri group that have a close size range, a similar number of kineties, or both (Table 1). More careful observations may be required in order to clearly distinguish these species from *E*. *curdsi*. *E*. *antarcticus* is a larger marine species (about 85 μm length) with 8 kineties that further differs from *E*. *curdsi* due to its peculiar shape (irregular and elongated, with a pointed posterior region) and 6 prominent dorsal ridges [51]. Its dargyrome type is also not easily discernible in the original description [37]. *E*. *magnicirratus* has 8 kineties and is approximately the same size as *E*. *curdsi*, but can be recognized by its broader shape, wide and extended peristome, 50 (vs. less than 35) membranelles in the adoral zone and the thick transverse cirri that give it its name [52]. *E*. *alatus* also has 8 kineties, but is considerably smaller (40 μm) and more rounded in appearance. Its peristome extends only about half of its body length, and the paroral membrane is tiny [53]. Also smaller (40 μm), but with the same number of kineties as *E*. *curdsi*, is *E*. *trisulcatus*, that can however be distinguished by a distinctly pointed posterior end, an apical notch and pronounced dorsal ridges [52, 54]. Finally, *E*. *balteatus* is remarkably polymorphic, with morphometric ranges so broad that they overlap with those of many other species. It is known to form giants and change size according to the feeding conditions [55], and further differs from *E*. *curdsi* because of its broader shape, wider peristome and inconspicuous paroral membrane [34].

**Table 1:** Morphological comparisons between *Euplotes curdsi* sp. nov. and five similar species. Characters that are unambiguously different are in bold.

	*E*. *curdsi* sp. nov.	*E*. *antarcticus*	*E*. *magnicirratus*	*E*. *alatus*	*E*. *trisulcatus*	*E*. *balteatus*
Size	45–65 μm	**85 μm**	51–65 μm	**36–43 μm**	35–50 μm	30–150 μm
Shape	Oval-ellipsoid; ends rounded	Elongated; **pointed posterior end**	Oval	Oval	Elongated; **pointed posterior end**	Oval
Peristome	~70% of the body; narrow	~75% of the body; narrow	~75% of the body; **wide**	**~50% of the body**	~65% of the body; narrow	~70% of the body; **wide**
Membranelles in the adoral zone	25–34	~30	**49–52**	~26	25–36	25–80
Dorsal ridges	5–6, inconspicuous	6, **prominent**	**Prominent**	Inconspicuous	**Prominent, 3 deep furrows**	3–5, **prominent**
Dargyrome type	Double-*eurystomus*	Double-*eurystomus* (?)	Double-*eurystomus*	Double-*eurystomus*	Double-*eurystomus*	Double-*eurystomus*
FVC pattern	10	10	10	10	10	10
Dorsolateral kineties	6–7	**8**	**8**	**8**	7	7–8
Dikinetids in mid-dorsal row	10–12	~13	**13–17**	10–12	11	10–16
Reference	This work	[51]	[52]	[53]	[52, 54]	[34]

FVC, frontoventral cirri.

The 18S rRNA genes of the five mentioned morphospecies have been sequenced, and they are all phylogenetically distant to *E*. *curdsi* (Fig 3), though it should also be noticed that these sequences are not accompanied by morphological descriptions (with the exception of a recently described population of *E*. *balteatus* [34]). Of the species in the same clade as *E*. *curdsi*, none can be confused morphologically with it. *E*. *nobilii*, *E*. *raikovi* and *E*. *elegans* have a double-*patella* dargyrome type [26, 56, 57]. The hypersaline *E*. *qatarensis* shares the double-*eurystomus* dargyrome type of *E*. *curdsi*, but has invariably 10 kineties, and an anterior projection on the right side of the body [36].

There are still a handful of named species in literature whose description lacks essential features, such as the dargyrome type [37]. It would normally be difficult to perform accurate comparisons with these taxa, but to our knowledge only two of them possess 10 frontoventral cirri. Firstly, *E*. *dammamensis* is much larger (more than 100 μm vs. 57 μm) than *E*. *curdsi* and the 18S rRNA gene of its type strain has been sequenced [34], allowing us to confirm that they are unrelated species (Fig 3). Secondly, *E*. *roscoffensis* is only slightly larger than *E*. *curdsi*, but the elevated number of membranelles in the adoral zone (up to 50) and the unique invagination of the peristome border supporting the paroral membrane [58] confirm that the two species cannot be the same.

### Environment and biogeography of the genus *Euplotes*

Ancestrally marine, the genus *Euplotes* has invaded freshwater and soil habitats during at least two radiations: within clade A, in the lineage of *E*. *muscorum* and allied species; and within clade B, before the lineages of *E*. *eurystomus*, *E*. *patella* and allied species diverged. *E*. *harpa* may have conserved the brackish-euryhaline status of an intermediate stage in the latter process. Other immigrations from marine to freshwater environments may have happened occasionally: an undescribed species related to the marine *E*. *elegans* was found in a freshwater marshland in Italy [50], and the only available sequence identified as *E*. *alatus*, traditionally considered marine, was obtained from a freshwater pond in Germany [49]. Moderate or extensive euryhalinity can be observed in several species (e.g. *E*. *euryhalinus* [57], *E*. *elegans* [26] and *E*. *woodruffi* [59]), and species of the *eurystomus*-*patella* freshwater radiation are routinely cultured at 5‰ salinity [18].

Recently, the two distantly related species *E*. *qatarensis* [36] and *E*. *dammamensis* [34] have been characterized from hypersaline locations, and it is plausible that others could be found by more extensive sampling of these environments. As a genus, *Euplotes* seems to be relatively prone to adaptation at different salinity levels, although the extent of this ability may vary with the clade. Clade B, that includes *E*. *curdsi*, seems particularly predisposed to tolerate such changes, since it includes taxa from all environments. *E*. *curdsi* itself is a euryhaline species found in both marine and brackish waters. Furthermore, the correlation between morphological change and salinity transition, for example the increases in size observed in clade B and for *E*. *dammamensis* following transition from marine to brackish and fresh, and hypersaline, respectively, suggests that *Euplotes* may be a useful model in studying salinity adaptations. However, especially in older literature salinity values may not be reported, and only descriptive environmental terms are used. As a consequence, it is likely that populations described as “marine” may have come from samples with low salinity.

Data are still too sparse to comment on the geographic distribution of most *Euplotes* species, or even subclades, with any degree of certainty. Geographic characters mapped in Fig 3 may very well be more reflective of the distribution of ciliate taxonomists than the actual prevalence of *Euplotes* species. However, a high level of cosmopolitanism is apparent for the genus and many of its subdivisions. Some of the individual morphospecies may perhaps be endemic of small areas, but most can be found in distant locations. Studies on fast-evolving markers will tell us more about the intraspecific relations among allopatric populations. As a testament to the adaptability of *Euplotes*, the invasion of cold polar waters and the consequent evolution of adaptive features [14, 15] has occurred no less than four independent times (Fig 3).

### Character evolution of the genus *Euplotes*

Size, number of kineties, number of membranelles in the adoral zone, structure of the nuclear apparatus, frontoventral cirri pattern and dargyrome type are the main traditional characters used in the identification of *Euplotes* species [37, 53]. Of these, the first three are to some degree correlated, and probably change on a small evolutionary scale. Nuclear features are often reported in characterizations, but they are in fact of little taxonomic value. With the conspicuous “T” shaped exception of *E*. *woodruffi*, the single macronucleus is almost always “C” or “3” shaped, with tiny morphological variations whose range, in our opinion, is scarcely greater than the intraspecific variability. This is evidenced by the differences observed even within monoclonal strains, and in multiple descriptions of the same species by different authors (see examples in Curds [37]), while other characters remain identical.

Frontoventral cirral pattern and dargyrome type are instead solid characters for classification, hardly changing within species and easily categorized. They were even used to split *Euplotes* into four different genera [60] in a system that, however, has been proved evolutionary inconsistent by molecular analysis [29, 36]. It is now known that the number of frontoventral cirri and the dargyrome type are not good characters for genus-level systematics, and the reasons are that: (1) the most common combination (10 FVC and the double-*eurystomus* pattern) is indeed a plesiomorphy retained by many lineages, and (2) the same changes happened independently multiple times.

#### Dargyrome evolution

The shift from the ancestral double-*eurystomus* dargyrome type to the double-*patella* type seems to be the most common (five or six independent occurrences), as the similarities between the two types would suggest (the opposite event might have happened once or never). It has been shown that the alveoli are not necessarily different in size in the two “double” types [61], but appear so in silver staining due to the degree of folding of the dorsal cortex, and Gates and Curds [62] even cast doubts on the reliability of this distinction for taxonomic purposes. Three other events are apparently lineage-specific: the transition from double-*eurystomus* to single (*vannus* subclade) and multiple (*muscicola* subclade) dargyrome types, and a successive transition from multiple to complex within the *muscicola* subclade (in *E*. *muscorum*), although the split between “multiple” and “complex” was also considered dubious by some [62]. Changes from the double dargyrome to other types are hence the result of multiplication [61] or fusion of alveoli. This evolutionary process is recapitulated in species with extreme phenotypic plasticity such as *E*. *balteatus* [55] and the “supergiant”-forming *E*. *versatilis* [63]. Since there is scant knowledge about the function of these structures, it’s impossible to speculate about potential adaptive reasons for these changes.

#### Frontoventral cirri evolution

The evolution of frontoventral cirri is instead a story of losses. Many *Euplotes* species have lost from one to three of the ten original cirri, as also evidenced by the vestiges still present in some species [26, 32, 64]. Little is known about the biomechanics of cirral locomotion, but the pattern shown would fit with non-adaptive losses due to drift, possibly following bottlenecks associated with speciation. Thanks to their somewhat rigid arrangement and ontogenesis in *Euplotes* it is possible to figure out which cirri are lost during each event. They vary with the lineage (and the difference is of taxonomic and systematic importance), but they are always the cirri closest to the median axis of the cell (2/II, 2/III, 2/V and 3/V). In turn, this consideration leads us to believe that the “external” frontoventral cirri fulfill a more essential role, or that their developmental mechanisms are more stable. The morphogenesis of cirri during cell division has been described in several species, and it is the only known developmental process that significantly varies within the genus [65].

#### Symbiosis and evolution in Euplotes

Changes in the dargyrome type and the number of frontoventral cirri do not seem to be strongly linked with each other or with environmental shifts in the inferred evolutionary history of the genus. An important evolutionary event that is clearly tied to phylogeny and habitat is instead the origin of symbiosis between *Euplotes* species and the bacterium *Polynucleobacter* (or other betaproteobacteria) [18, 19, 66, 67]. This obligate relationship, explored in many details but of still mysterious function, was clearly made possible by the invasion of the bacteria’s habitat by *Euplotes* species. Interestingly, while all brackish and freshwater populations of *E*. *harpa* screened to date possess the endosymbiont nothing is known about marine populations or *E*. *neapolitanus*, only described from sea samples [68]. This missing information is rather crucial, since it is unlikely that the freshwater *Polynucleobacter* could be associated with marine organisms. More generally, bacterial symbioses in marine *Euplotes* are less known, though still reported [69, 70]. This is another instance where it is unclear if the feature reflects an occurrence pattern, or biases in symbiosis researchers focusing on freshwater species.

In a similar manner, symbioses between *Euplotes* and other eukaryotes have not been studied systematically, but *E*. *daidaleos* and *E*. *uncinatus* are known to harbor potentially beneficial endocellular algae [30, 71], while other species may be hosts of parasites such as microsporidia [59] and trypanosomes [72].

**Phylum Ciliophora Doflein**, **1901**

**Class Spirotrichea Bütschli**, **1889**

**Subclass Euplotia Jankowski**, **1979**

**Order Euplotida Small and Lynn**, **1985**

**Family Euplotidae Ehrenberg**, **1838**

**Genus Euplotes Ehrenberg**, **1831**

***Euplotes curdsi* sp. nov**.

### Diagnosis

Medium-small (45–65 μm) *Euplotes* species. Shape oval to elongated, with both ends rounded. No anterior notch or peristomial collar. Inconspicuous ridges on dorsal side, three deep ridges on ventral side. Peristome narrow and deep. Conspicuous paroral membrane. Cytostome around 65–75% of body length. 25–34 membranelles in the adoral zone. Dorsal argyrome of the double-*eurystomus* type, with 6–7 kineties and 10–12 dikinetids in the mid-dorsal row. 10 frontoventral, 5 transverse and 2–3 caudal cirri (including 2 marginal cirri on the left side of the cell). Contractile vacuole often not observed. Macronucleus variable, usually “C” or “3” shaped, with irregularly dense chromatin. Single micronucleus strictly associated with the macronucleus in anterior position.

Reported from brackish and marine samples in the Mediterranean Sea and White Sea. No symbiont known.

### Type locality

Brackish Orbetello Lagoon (Grosseto, Tuscany, Italy). 42°26’15” N, 11°11’39 E.

### Type material

Slides with silver nitrate stained cells of the type strain Min1 are deposited at the Beaty Biodiversity Museum (University of British Columbia, Vancouver, Canada; accession numbers: A92521-2). The SSU rRNA gene sequence is deposited in GenBank (accession number: LT615048). A living culture is available at the Culture Collection of Algae and Protozoa (CCAP 1624/28).

### Etymology

The species is named after Colin R. Curds, author of an extensive review on the *Euplotes* genus (Curds 1975).

## Supporting Information

S1 Fig Maximum Likelihood phylogenetic tree of the genus *Euplotes* based on SSU rRNA gene sequences (164-sequence dataset, 2162 characters), formatted as in Fig 3. The root was placed between *Euplotes* sequences and the outgroup. Purple dots are associated to nodes that differ between the trees inferred from the two datasets. The only such node receiving any statistical support is the one clustering *E*. *quinquecarinatus* with *E*. *charon* (JF694043) and *E*. *parkei* (bootstrap: 78%; posterior probability: 1.00). *E*. *quinquecarinatus* is instead the sister species of *E*. *focardii* in the 46-sequence dataset (low support). Morphospecies with more than one sequence are mostly monophyletic, with the exception of *E*. *charon* (discussed in the text) and *E*. *encysticus* (in an unresolved clade with *E*. *novemcarinatus*). *E*. *euryhalinus* (split into two divergent groups) and *E*. *daidaleos* appear monophyletic, but with low support.
(PDF)
