# Supplementary material for: Biogeography and Character Evolution of the Ciliate Genus Euplotes (Spirotrichea, Euplotia), with Description of Euplotes curdsi sp. nov
Source: PLoS One. 2016 Nov 9;11(11):e0165442. doi: 10.1371/journal.pone.0165442 (PMC5102374; doi:10.1371/journal.pone.0165442)
Supplement: S1 Fig — The root was placed between Euplotes sequences and the outgroup. Purple dots are associated to nodes that differ between the trees inferred from the two datasets. The only such node receiving any statistical support is the one clustering E. quinquecarinatus with E. charon (JF694043) and E. parkei (bootstrap: 78%; posterior probability: 1.00). E. quinquecarinatus is instead the sister species of E. focardii in the 46-sequence dataset (low support). Morphospecies with more than one sequence are mostly monophyletic, with the exception of E. charon (discussed in the text) and E. encysticus (in an unresolved clade with E. novemcarinatus). E. euryhalinus (split into two divergent groups) and E. daidaleos appear monophyletic, but with low support. (PDF) [file pone.0165442.s001.pdf]

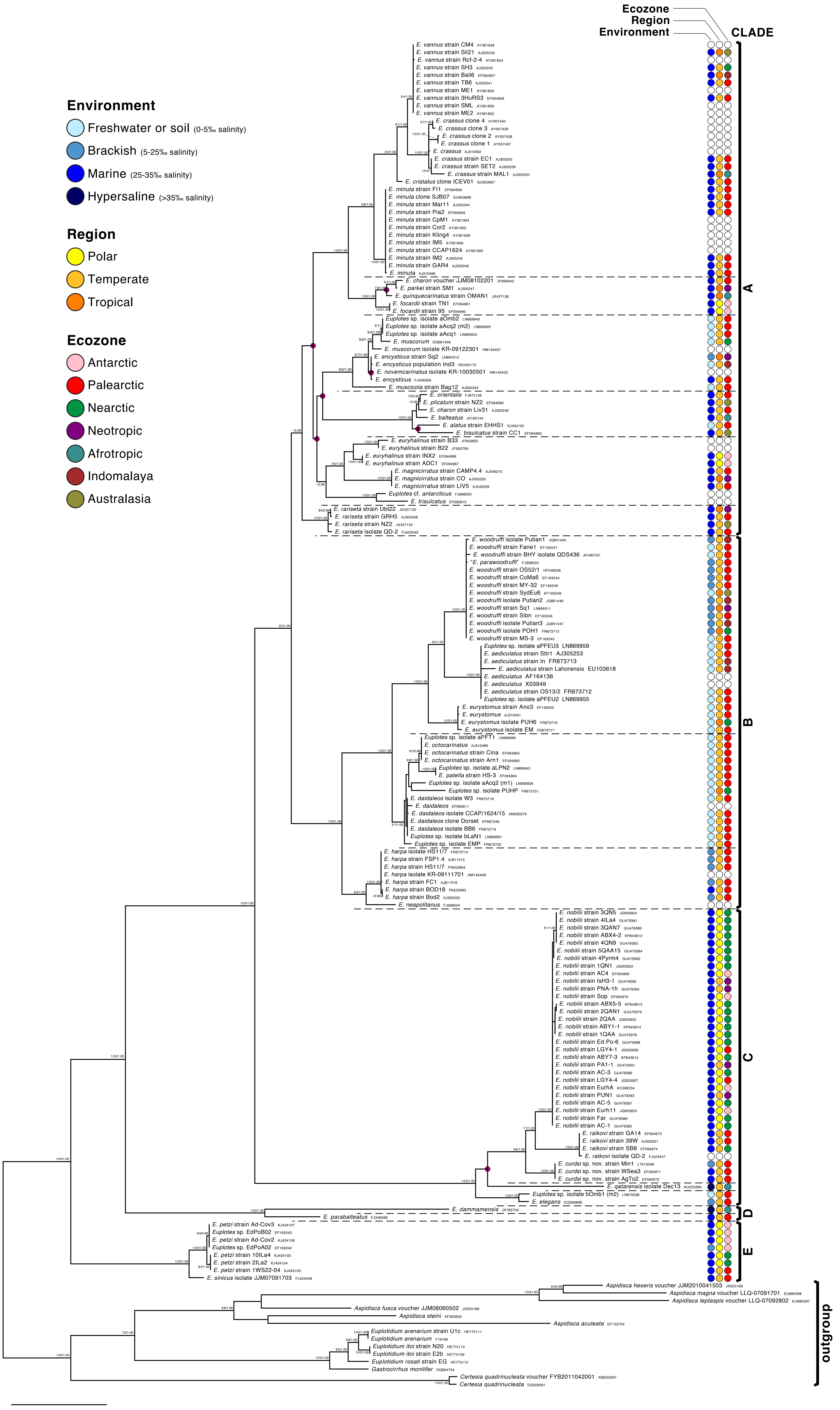

Ecozone
Region
Environment
CLADE
Environment
Freshwater or soil (0-5‰ salinity)
Brackish (5-25‰ salinity)
Marine (25-35‰ salinity)
Hypersaline (>35‰ salinity)
Region
Polar
Temperate
Tropical
Ecozone
Antarctic
Palearctic
Nearctic
Neotropic
Afrotropic
Indomalaya
Australasia
A
B
C
D
E
outgroup
E. vannus strain CM4
E. vannus strain Sil21
E. vannus strain Rcf-2-4
E. vannus strain SH3
E. vannus strain Bali6
E. vannus strain TB6
E. vannus strain ME1
E. vannus strain 3HuRS3
E. vannus strain SML
E. vannus strain ME2
E. crassus clone 4
E. crassus clone 3
E. crassus clone 2
E. crassus clone 1
E. crassus
E. crassus strain EC1
E. crassus strain SET2
E. crassus strain MAL1
E. cristatus clone ICEV01
E. minuta strain FI1
E. minuta clone SJB07
E. minuta strain Mar11
E. minuta strain Pia2
E. minuta strain CpM1
E. minuta strain Cor2
E. minuta strain Kling4
E. minuta strain IM5
E. minuta strain CCAP1624
E. minuta strain IM2
E. minuta strain GAR4
E. minuta
E. charon voucher JJM08102201
E. parkei strain SM1
E. quinquecarinatus strain OMAN1
E. focardii strain TN1
E. focardii strain 95
Euplotes sp. isolate aOmb2
Euplotes sp. isolate aAcq2 (m2)
Euplotes sp. isolate aAcq1
E. muscorum
E. muscorum isolate KR-09122301
E. encysticus strain Sq2
E. encysticus population Ind3
E. novemcarinatus isolate KR-10030501
E. encysticus
E. muscicola strain Bag12
E. orientalis
E. plicatum strain NZ2
E. charon strain Liv31
E. balteatus
E. alatus strain EHHS1
E. bisulcatus strain CC1
E. euryhalinus strain B33
E. euryhalinus strain B22
E. euryhalinus strain INX2
E. euryhalinus strain ADC1
E. magnicirratus strain CAMP4.4
E. magnicirratus strain CO
E. magnicirratus strain LIV5
Euplotes cf. antarcticus
E. trisulcatus
E. rariseta strain Ubt22
E. rariseta strain GRH5
E. rariseta strain NZ2
E. rariseta isolate QD-2
E. woodruffi isolate Putian1
E. woodruffi strain Fane1
E. woodruffi strain BHY isolate QDS436
"E. parawoodruffi"
E. woodruffi strain OS52/1
E. woodruffi strain CoMa6
E. woodruffi strain MY-32
E. woodruffi strain SydEu6
E. woodruffi isolate Putian2
E. woodruffi strain Sq1
E. woodruffi strain Sibn
E. woodruffi isolate Putian3
E. woodruffi isolate POH1
E. woodruffi strain MS-3
Euplotes sp. isolate aPFEU3 LN869959
E. aediculatus strain Stir1 AJ305253
E. aediculatus strain In FR873713
E. aediculatus strain Lahorensis EU103618
E. aediculatus AF164136
E. aediculatus X03949
E. aediculatus strain OS13/2 FR873712
Euplotes sp. isolate aPFEU2 LN869955
E. eurystomus strain Ano3
E. eurystomus
E. eurystomus isolate PUH6
E. eurystomus isolate EM
Euplotes sp. isolate aPFT1
E. octocarinatus
E. octocarinatus strain Cina
E. octocarinatus strain Arn1
Euplotes sp. isolate aLPN2
E. patella strain HS-3
Euplotes sp. isolate aAcq2 (m1)
Euplotes sp. isolate PUHP
E. daidaleos isolate W3
E. daidaleos
E. daidaleos isolate CCAP/1624/15
E. daidaleos clone Dorset
E. daidaleos isolate BB6
Euplotes sp. isolate bLaN1
Euplotes sp. isolate EMP
E. harpa isolate HS11/7
E. harpa strain FSP1.4
E. harpa strain HS11/7
E. harpa isolate KR-09111701
E. harpa strain FC1
E. harpa strain BOD18
E. harpa strain Bod2
E. neapolitanus
E. nobilii strain 3QN5
E. nobilii strain 4ILa4
E. nobilii strain 3QAN7
E. nobilii strain ABX4-2
E. nobilii strain 4QN9
E. nobilii strain 5QAA15
E. nobilii strain 4Pyrm4
E. nobilii strain 1QN1
E. nobilii strain AC4
E. nobilii strain IsH3-1
E. nobilii strain PNA-1h
E. nobilii strain Sop
E. nobilii strain ABX5-5
E. nobilii strain 2QAN1
E. nobilii strain 2QAA
E. nobilii strain ABY1-1
E. nobilii strain 1QAA
E. nobilii strain Ed.Po-6
E. nobilii strain LGY4-1
E. nobilii strain ABY7-3
E. nobilii strain PA1-1
E. nobilii strain AC-3
E. nobilii strain LGY4-4
E. nobilii strain EurhA
E. nobilii strain PUN1
E. nobilii strain AC-5
E. nobilii strain Eurh11
E. nobilii strain Far
E. nobilii strain AC-1
E. raikovi strain GA14
E. raikovi strain 39W
E. raikovi strain SB8
E. raikovi isolate QD-2
E. curdsi sp. nov. strain Min1
E. curdsi sp. nov. strain WSea3
E. curdsi sp. nov. strain AgTo2
E. qatarensis isolate Dec13
Euplotes sp. isolate bOmb1 (m2)
E. elegans
E. dammamensis
E. parabalteatus
E. petzi strain Ad-Cov3
Euplotes sp. EdPoB02
E. petzi strain Ad-Cov2
Euplotes sp. EdPoA02
E. petzi strain 10ILa4
E. petzi strain 2ILa2
E. petzi strain 1WS22-04
E. sinicus isolate JJM07091703
Aspidisca hexeris voucher JJM2010041503
Aspidisca magna voucher LLQ-07091701
Aspidisca leptaspis voucher LLQ-07092802
Aspidisca fusca voucher JJM08060502
Aspidisca steini
Aspidisca aculeata
Euplotidium arenarium strain U1c
Euplotidium arenarium
Euplotidium itoi strain N20
Euplotidium itoi strain E2b
Euplotidium rosati strain EG
Gastrocirrhus monilifer
Certesia quadrinucleata voucher FYB2011042001
Certesia quadrinucleata
